# Priming Treatments with Biostimulants to Cope the Short-Term Heat Stress Response: A Transcriptomic Profile Evaluation

**DOI:** 10.3390/plants11091130

**Published:** 2022-04-21

**Authors:** Giacomo Cocetta, Michela Landoni, Roberto Pilu, Carlos Repiso, José Nolasco, Marcos Alajarin, Lydia Ugena, Camila C. B. Levy, Giacomo Scatolino, Daniele Villa, Antonio Ferrante

**Affiliations:** 1 DISAA—Department of Agricultural and Environmental Sciences, Università degli Studi di Milano, Via Celoria 2, 20133 Milano, Italy; salvatore.pilu@unimi.it (R.P.); antonio.ferrante@unimi.it (A.F.); 2 Department of Bioscience, Università degli Studi di Milano, Via Celoria 26, 20133 Milano, Italy; michela.landoni@unimi.it; 3 Tradecorp International, Via de los Poblados, 3. Edif. Onic 5, 6th Floor, 28033 Madrid, Spain; carlesrepiso@gmail.com (C.R.); jose.nolasco@tradecorp.rovensa.com (J.N.); alajarin.marcos@gmail.com (M.A.); lydia.ugena@tradecorp.rovensa.com (L.U.); camila.levy@tradecorp.rovensa.com (C.C.B.L.); 4 Agricola 2000, scpa Via Trieste 9, 20067 Tribiano, MI, Italy; g.scatolino@agricola2000.com (G.S.); d.villa@agricola2000.com (D.V.)

**Keywords:** abiotic stress, *Arabidopsis thaliana*, *Ascophyllum nodosum*, HPS, heat shock, secondary metabolism, high temperature

## Abstract

Plant stress induced by high temperature is a problem in wide areas of different regions in the world. The trend of global warming is going to enhance the effects of heat stress on crops in many cultivation areas. Heat stress impairs the stability of cell membranes and many biological processes involving both primary and secondary metabolism. Biostimulants are innovative agronomical tools that can be used as a strategy to counteract the detrimental effect of abiotic stresses, including heat stress. In this work, two biostimulants based on *Ascophyllum nodosum* extracts (named Phylgreen) and based on animal L-α amino acids (named Delfan Plus) were applied as priming treatments to *Arabidopsis thaliana* plants subjected to heat stress exposure. Plants at the vegetative stage were treated with biostimulants 12 h before high temperature exposure, which consisted of maintaining the plants at 37 ± 1 °C for 4 h. Transcriptional profiles, physiological, and biochemical analyses were performed to understand the mode of action of the biostimulants in protecting the plants exposed to short-term heat stress. At a physiological level, chlorophyll, chlorophyll a fluorescence, phenolic index, total anthocyanins, reactive oxygen species (ROS) were measured, and significant variations were observed immediately after stress. Both biostimulants were able to reduce the oxidative damage in leaves and cell membrane. Transcriptomic data revealed that upregulated genes were 626 in Phylgreen and 365 in Delfan Plus, while downregulated genes were 295 in Phylgreen and 312 in Delfan Plus. Bioinformatic analysis showed that the biostimulants protected the plants from heat stress by activating specific heat shock proteins (HPS), antioxidant systems, and ROS scavengers. The results revealed that the biostimulants effectively induced the activation of heat stress-associated genes belonging to different transcription factors and HSP families. Among the heat shock proteins, the most important was the AtHSP17 family and in particular, those influenced by treatments were AtHPS17.4 and AtHPS17.6A, B, showing the most relevant changes.

## 1. Introduction

Heat stress is well known as one of the main abiotic stresses that affect the performance of cultivated crops, and its potential severity is going to be increased by global climate change [1]. Heat stress due to high temperature can negatively affect plant growth, development, and, more severely, the reproductive stages causing a decrease of crop yield [2]. The exposure of crops to heat stress is not always constant and its intensity generally shows a gradual increase until the early afternoon and then decline until the end of the day. Moreover, different weather conditions can affect heat stress intensity with daily changes. This behavior is commonly known as a heat wave and it can influence crop performance during the summer season [3]. The main consequence of stress conditions produced by high temperatures is the cell membrane damage which leads to the loss of cellular organization and to cell death [4]. Moreover, plant injuries include protein denaturation, aggregation, and enhanced fluidity of membrane lipids. High temperatures also affect the organization of microtubules. The severity of damage depends on the time of exposure and the temperature intensity. All this damage is due to the loss of cell compartmentalization that induces the activation and loss of enzymatic activities, ion flux disorder, accumulation of toxic compounds, including reactive oxygen species (ROS) [4]. The reproductive growth is altered by heat stress in various species, including rice, which showed an optimum at 33 °C for vegetative growth, while grain formation and yield were negatively affected by temperatures above 25 °C. Moreover, temperatures above 33 °C reduced the viability of pollen which reached zero for temperatures of 40 °C with a similar phenomenon for sorghum (optimum at 26–34 °C for vegetative growth and at 25–28 °C for reproductive growth) and for *Arabidopsis thaliana*, in which the abortion of the whole inflorescence was observed at a temperature of 36 °C [5]. The same authors emphasized the cellular response to temperature stress which includes the altered organization of organelles, cytoskeleton, and membrane structures. To maintain membrane stability and normal cellular functions in the presence of heat stress, plants synthesize heat shock proteins (HSPs), molecular chaperones that prevent protein misfolding or aggregation, as well as other co-chaperones, hormones, and other protective molecules. These HSPs have a role in protein quality control and can protect the protein functionality, also under stress [6]. Their function in plants is essential for normal growth and development, this can explain their activation, also under heat stress responses in order to guarantee plant performance. The expression of genes encoding for the HSPs is induced by heat stress transcription factors (HSFs) that bind to heat shock elements in the promoters of HSPs. There are many steps of regulation allowing dynamic control of the heat stress response, as the HSFs themselves can be post-transcriptionally modified. In addition to the constitutive role that HSPs play in heat stress responses across cell types, these proteins can acquire specialized functions that regulate developmental responses of organs to environmental stress [7,8].

The expression of a variety of biochemical markers correlates plant responses to abiotic stress. Secondary metabolites, and in particular phenolic compounds, playing a major role in the adaptation of plants to the environment and in overcoming stress conditions, can be easily detected because of their natural autofluorescence. Environmental factors such as air temperature and humidity, light intensity, water supply, mineral nutrition, and CO_2_ influence plant growth and secondary metabolite production [9].

HSPs are clustered into five families highly evolutionarily conserved as small heat stress proteins, (sHPSs < 40 kDa) HSP60, HSP70, HSP90, HSP100. The HSP100 family is not exclusively expressed under heat stress but also under other abiotic stresses such as salinity, drought, abscisic acid, and cold stress. HSP90 genes have been found in different plants exposed to various abiotic stresses such as sub-optimal temperature, salinity, and heavy metals [4]. The HSP90 protein is required both for normal development and stress tolerance. It represents 1–2% of total proteins in unstressed cells and is involved in many cellular processes. The role of HSP includes the stabilization and regulation of homeostasis of proteins, transcriptional regulation, chromatin organization, defence mechanisms, and DNA repair. HSP70 proteins are molecular chaperones and found in almost all plant species. The sHSPs are a large family of proteins that are not normally expressed in plants under optimal conditions but increase if exposed to stress conditions.

Hydrogen peroxide is one of the main ROS and it is known to play a central role in plant responses to abiotic stresses. An excessive production and accumulation of ROS in plant tissues induces oxidative stress. The ROS accumulation occurs in plants exposed to abiotic stress conditions [10]. Such oxidative stress, potentially damaging to plant cells, was reported in plants subjected to drought, flooding, high light intensity, chilling, heat, salinity, air pollutants, and herbicides. Enhanced ROS generation has also been found in heat stressed plants with an increase of membrane lipid peroxidation [11]. 

H_2_O_2_ is a stable ROS that has a pivotal role in response and defence in plants. The H_2_O_2_ is the regulator of physiological and biochemical processes in plants under biotic and abiotic stresses [12]. At high concentrations, it can be toxic, while in low concentrations, it activates specific defence mechanisms in plants, allowing them to overcome stressful conditions [13]. 

Heat stress is a common abiotic stress during summer in Mediterranean areas and the severity of the stress is increased if associated with low water availability. Prediction of these events is not easy with the available forecast models. Thus, farmers need additional practical tools to alleviate crops from abiotic stresses [14,15,16]. To date, several biostimulant products are available on the market and they can be used as a good agronomic tool to prepare plants for incoming critical periods (priming) or to counteract the negative effects and help plants to recover after stress [15,17]. Priming is an agronomic strategy used in agriculture for inducing the seeds or plants to activate specific physiological or biochemical processes for improving germination or plant performance against specific events (i.e., germination for seeds, counteract abiotic stresses for plants, etc.). Biostimulants can act by increasing the capacity of the crops to face a stressful condition and avoid the negative effects on their performance. This priming effect is due to the activation of a complex mechanism of molecular interactions [18] that is triggered by the active components of the biostimulant [19] (Fleming et al., 2018). Previous studies have shown that *Ascophyllum nodosum* seaweed extracts can act as biostimulants maintaining crop productivity during and after exposure to abiotic stresses, by protecting the plant cells from the stress-induced oxidative damage [20]. However, it has been shown that, starting from the same raw material, the success of a seaweed-based biostimulant depends on the manufacturing procedures [21]. Biostimulants containing amino acids can also be used as a priming treatment for the activation of different physiological processes. The application of amino acid-based biostimulants (Delfan Plus) applied as a priming treatment was able to increase drought tolerance, mitigating stress, in the *Arabidopsis thaliana* model [19]. Understanding the mechanism of action of biostimulants and the plant response to their application in combination with abiotic stress conditions is crucial for the optimization and use of these novel agronomic tools [22]. 

The aim of the work was to evaluate the priming effect of the biostimulant products applied to *Arabidopsis thaliana* plants subjected to a short-term heat stress (37 °C). This was achieved by the identification of the transcriptional changes in stressed plants previously treated with biostimulants. The physiological, biochemical, and molecular approaches provided useful information for understanding the mode of action of the products when applied prior to the stress (priming/preventive effect). The hypothesis of this work was that biostimulants containing *Ascophyllum nodosum* extracts or containing amino acids could be applied as protective treatments against heat stress in plants. Biochemical and molecular analyses have been used to identify the activation of the specific clusters of genes that are responsible for the increase in plant tolerance and recovery in plant performance after stress exposure.

## 2. Results

### 2.1. Transcriptional Changes

The RNA-seq analysis enabled identification of a total of 32,833 sequences corresponding to gene sequences in the Arabidopsis thaliana reference genome. Based on the count of each read, the expression of each gene was estimated as Log FC, compared to non-stressed control (CTR). The Venn diagram has been used for identifying the differentially expressed genes activated or repressed by the treatments. A total of 1756 DEGs were expressed under heat stress, while 1228 DEGs were found in response to Phylgreen application, and 844 in Delfan Plus application. Biostimulant treatments shared 1620 DEGs and only 174 were commonly expressed in the biostimulants and stress conditions (Table 1).

**Figure 1:**
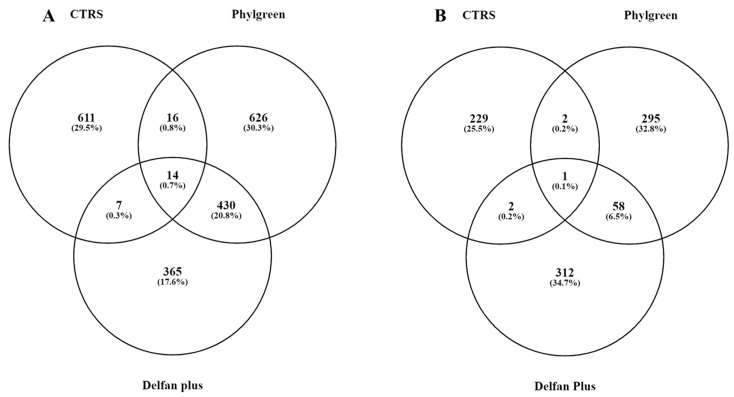
Common and specific differentially expressed genes (FC > 2) in stressed control (CTRS) and treated plants (Phylgreen and Delfan Plus) grouped by the Venn diagram. (**A**) Upregulated DEGs and (**B**) downregulated DEGs.

The DEGs with a fold change (FC) > 2 and up- or downregulated genes are shown in the Venn diagram (Figure 1A,B). Upregulated DEGs were 611 genes in CTRS, 626 genes in Phylgreen, and 365 in Delfan Plus (Figure 1A), 14 were DEGs in all treatments compared to CTR. Downregulated DEGs were 229 in CTRS, 295 in Phylgreen, 312 in Delfan Plus, only one gene was common in all treatments (Figure 1B). 

**Figure 2:**
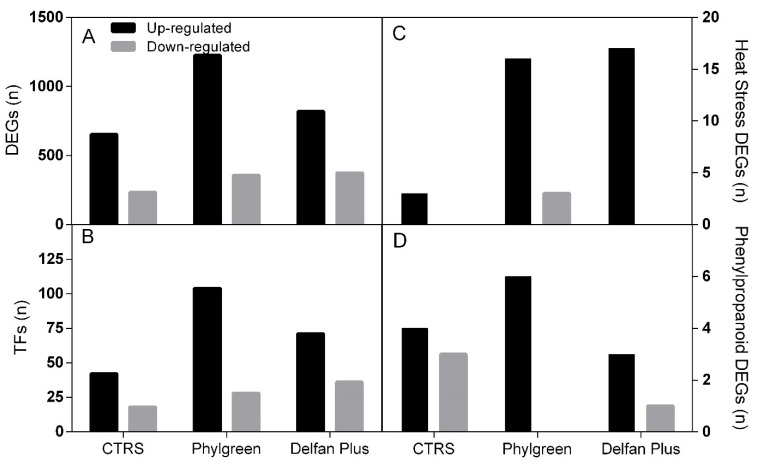
Total differently expressed genes (**A**), transcription factors (TFs) (**B**), heat stress-associated genes (**C**), phenylpropanoids (**D**) in CTRS, Phylgreen, and Delfan Plus.

The two biostimulants had different effects on transcription activation. Phylgreen and Delfan Plus showed a higher number of upregulated DEGs than downregulated. The total number of DEGs was higher in Phylgreen compared to the stressed control and Delfan Plus (Figure 2A, Supplementary Dataset S1).

### 2.2. Expression of Transcription Factors

Transcription factors are important regulators of gene expression, and their regulatory function is extremely important under stress conditions. Phylgreen and Delfan Plus showed changes in the number of transcription factors that were found differentially expressed compared to the control. In plants treated with Delfan Plus the upregulated genes were 71, while 36 were downregulated (Figure 2B). Only one gene, the *at2g38250* that encodes for Homeodomain-like superfamily protein was in common among the treatments. Phylgreen and Delfan Plus shared 35 differentially expressed transcription factors. The transcription factors mostly affected by Phylgreen and Delfan Plus (Log FC > 5) belonged to the families MYB, bHLH, and DREB which are usually involved in abiotic stress plant responses. The *AtbHLH* and *AtMYB1* genes regulate the activation of the shikimate pathway and were highly expressed in response to both biostimulants (Supplementary Dataset S2). The analysis of transcription activation between CTRS vs. Phylgreen showed that the *ZINC FINGER PROTEIN 6 (AtZEP2, AtZFP6), LITTLE ZIPPER 2 (AtZPR2), zinc finger, AP2 domain-containing transcription factor*, and *WRKY74* were upregulated in the Phylgreen compared to CTRS (Supplementary Dataset S1). The transcription activation in Delfan Plus compared to CTRS revealed that the transcription factors activated in plants under stress treated with Delfan Plus were *AtWRKY72, WRKY31, ALCATRAZ* (at5g67110), and *CHP-rich zinc finger protein putative* (Supplementary Data S1).

### 2.3. Heat Stress Response-Associated Genes

The analysis of genes specifically associated to heat stress response revealed a significant increment in the number of genes activated by all the treatments (stressed control and biostimulants) compared to untreated plants. In general, the biostimulants induced the overexpression of genes rather than the downregulation, as shown in Figure 2C.

The expression pattern observed in all treatments was similar and markedly different compared to the one of CTR. However, specific responses were induced by each of the treatments. All treatments induced or inhibited the expression (Log FC > 2 or < −2) of a few genes in a specific manner. Most of these genes encoded for heat shock proteins (HSP), which could be further used as marker for studying the specific mode of action of each biostimulant. In the stress-control (CTRS), the highest expressed genes were *AtHSP17.6*, HSP binding protein (at4g24190), and *AtHSP90.7*. The higher gene expression belonging to associated heat stress genes was found in the *HPS17* families (*AtHPS17.4, AtHPS17.6A, B*), between Phylgreen and Delfan Plus treatments. Downregulated genes in the biostimulants treatments were *AtBIP3* and heat shock-related protein (*At2g03020*). 

### 2.4. Secondary Metabolism—Phenylpropanoid Pathway

The DEGs encoding for secondary metabolism were analyzed, and differences were observed in the phenylpropanoid pathway. Delfan Plus treatment based on RNAseq data after 16 h showed a downregulation of this biosynthetic route, while no difference was observed as a response to Phylgreen application (Figure 2D). In CTRS, the *AtACOS5* (*Acyl-COA Synthetase 5*) and *4-coumarate-CoA ligase genes* were upregulated, while *laccase/diphenol oxidase* and transferase family protein were downregulated. In Phylgreen treated plants, *AtCCR2*, (cinnamoyl-CoA reductase), O-methyltransferase family 2 protein, and 4-coumarate-CoA ligase family protein were upregulated. No downregulated genes with FC < −2 were observed (Supplementary Dataset S2).

Delfan Plus application induced the expression of genes that encode for *UDP-glycosyltransferase/coniferyl-alcohol glucosyltransferase/transferase (UGT72E2), cinnamoyl-CoA reductase-related*, and an O-methyltransferase family 2 protein (*at1g77530*), while only downregulating one gene (Supplementary Dataset S2). This gene was encoding for an O-methyltransferase family 2 protein (*at4g35160*).

A mitochondrial ***At****HSP23-5* was upregulated in both biostimulants ranging from 3.4 to 4.3 FC. This gene could play an important role in heat stress tolerance. 

### 2.5. DAVID Enrichment Analysis

The enrichment analysis was performed to identify classes of genes that were over-represented in each treatment and an association with a specific biostimulant treatment could be highlighted. The results obtained from the enrichment analyses are shown as functional annotation clustering and gene function classification (Supplementary Dataset S3).

In CTRS, the enrichment analysis showed a fold enrichment > 1.0^15^ and the top ten categories highlighted in CTRS treatments were SAMRT, INTERPRO, GOTERM, KEGG_PATHWAY, and UP_SEQ_FEATURE (Table 2). The most significant terms enriched were the knottin, a scorpion toxin-like, palmitoyl protein thioesterase in the INTERPRO category. The thiol-ester and palmitoyl hydrolase activity were the most enriched terms in the GOTERM category. In the Phylgreen treatment the top ten categories were the same as CTRS except for the KEGG_PATHWAY and the most enriched terms were related to wounding response, following from AP2/ERF DNA binding protein. Delfan Plus treatment showed the GOTERM, UP_SEQ_FEATURE, and UP_Keywords as the most enriched categories, which include abiotic defence response and oxidoreductase or oxidation-reduction process. 

The DAVID functional annotation clustering (FAC) considering an Enrichment Score > 2 identified one cluster for CTRS, 11 for Phylgreen (Supplementary Data S3) and two for Delfan Plus. The FAC analysis reported for the CTRS terms mainly belonged to fatty acid metabolism (Table 3). The two highest enriched clusters identified in the Phylgreen treatment included transcriptional regulation terms and ethylene signalling pathways (Table 3). The first class with an enrichment score of 5.5 included ROS, heat, and high light responsive genes. The second enrichment score of 4.5 included the WRKY transcription factor in the following terms DNA-binding region: WRKY, SM00774:WRKY, and IPR003657:DNA-binding WRKY. The Phylgreen showed the highest percentage of genes annotated in cluster 2 and with percentages ranging from 5 to 11.6% (Table 3). 

### 2.6. Histochemical Analysis

**Figure 3:**
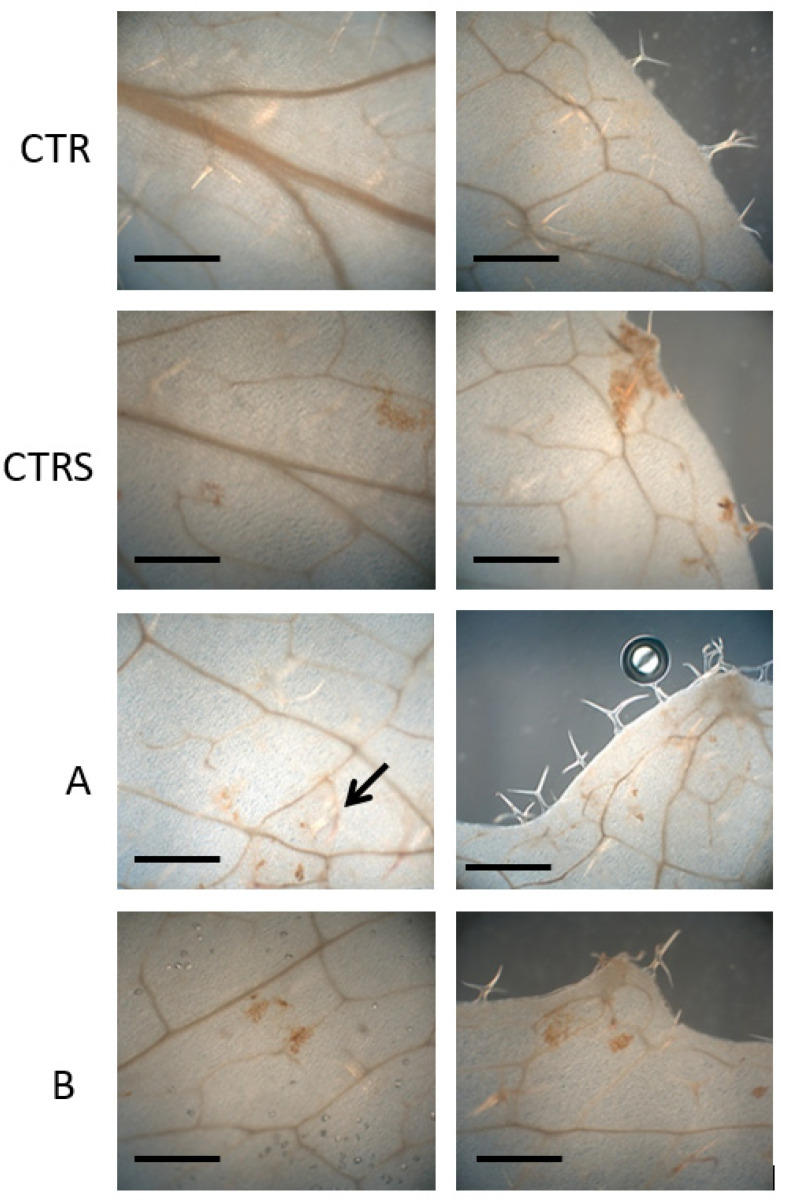
ROS histolocalization by DAB staining on mature leaves. The brown spots represent the sites where ROS are accumulated. For each sample, one picture was taken from the central area of the leaf blade (**left**) and one from the leaf border (**right**). CTR: non-stressed control, CTRS: stressed control, A: Phylgreen, B: Delfan Plus. Bars: 500 µm. The arrow indicates the faint signal caused by treatment drops.

**Figure 4:**
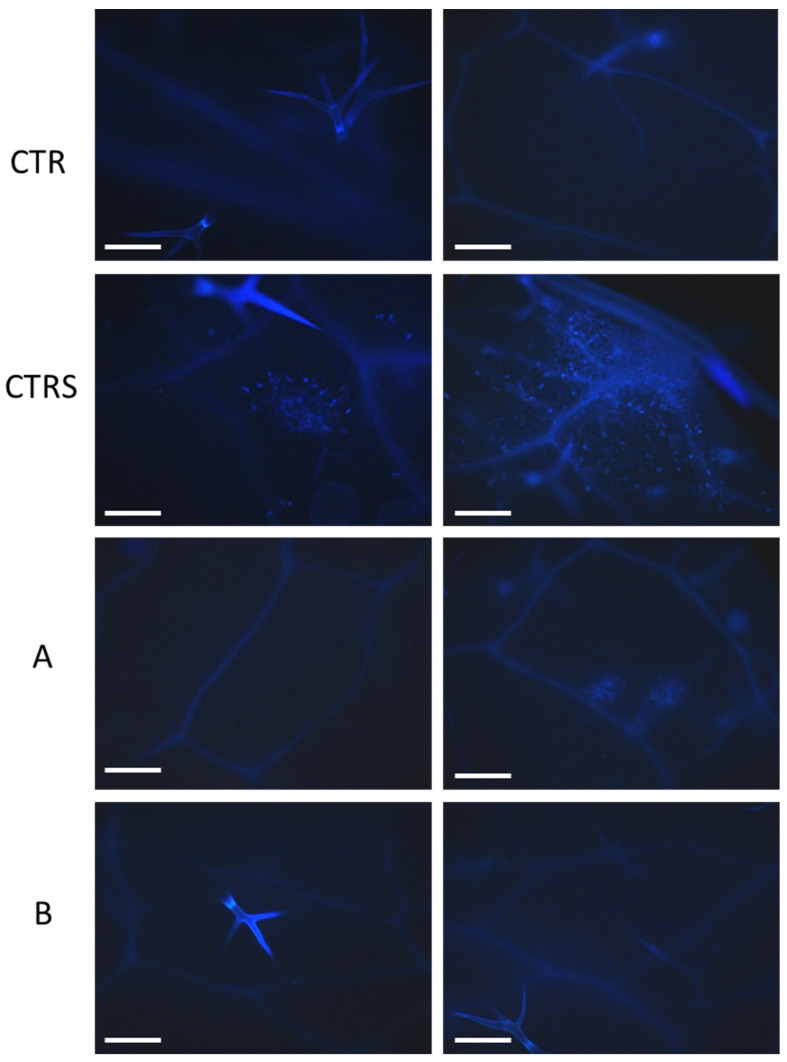
Phenylpropanoid histolocalization by autofluorescence visualization. For each sample one picture was taken from the central area of the leaf blade (**left**) and one from the leaf border (**right**). CTR: non-stressed control, CTRS: stressed control, A: Phylgreen, B: Delfan Plus. Bars: 200 μm.

For all the samples, the presence of secondary metabolites of the phenylpropanoid pathway (mainly lignin) and ROS (H_2_O_2_) by histochemical analysis on mature leaves, in non-stressed control (CTR), stressed control CTRS, and treated plants were analyzed. ROS were visualized by 3,3’-diaminobenzidine (DAB) staining. DAB is oxidized in the presence of peroxidase and hydrogen peroxide resulting in the deposition of a brown, alcohol-insoluble precipitate at the site of enzymatic activity (Figure 3). The CTR did not show any brown spots. The stressed control CTRS showed several brown spots along the leaf margins and spread onto the leaf blade, indicating the accumulation of ROS in different areas of the leaves. Phylgreen and Delfan Plus application strongly reduced the ROS accumulation and only a few small spots were visible, demonstrating the positive effects of these treatments against heat stress (Figure 4). In particular, leaves treated with Phylgreen (A) and Delfan Plus (B) only showed small patches of DAB signal along the leaf margins or on the leaf blade. It is interesting to note that on the leaf areas reached by the drops of the products Phylgreen and Delfan Plus used to treat the plants, a faint but clear DAB signal was present. Damaged cells induced by heat stress could be visualized using autofluorescence. The intensity of autofluorescence depends on the cell wall composition and construction. Changes in autofluorescence can be used only as a coarse indicator of cell wall products. High autofluorescence signals were found in CTRS, indicating the heat stress damage. In contrast, no damage was found in control plants, while almost no autofluorescence signals were found in two samples treated with Delfan Plus (B) and only a few spots were found in leaves treated with Phylgreen (A) (Figure 4).

**Figure 5:**
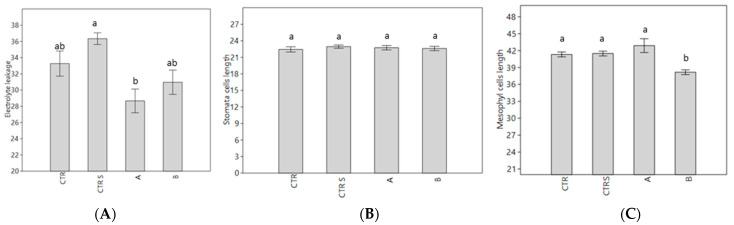
Electrolyte leakage expressed as μS/cm (**A**), stomata cell length in μm (**B**), and mesophyll cells in μm, (**C**). On the x-axis, CTR, CTRS, A: Phylgreen, B: Delfan Plus. Means followed by the same letter are not significantly different (Tukey test, *p* < 0.05). Data are means of three biological replicates (*n* = 50). Bars represent standard error.

The cell membrane breakdown was estimated by measuring the electrolyte leakage. The results indicated that plants treated with Phylgreen showed lower values than CTR, CTRS, and Delfan Plus. The electrolyte leakage results demonstrated that Phylgreen preserved the membrane integrity even under heat stress conditions (Figure 5). The Delfan Plus treatment showed similar membrane integrity to the controls (CTR and CTRS). The stomata cell length and mesophyll cells were affected by neither the biostimulants nor the heat stress. 

### 2.7. Physiological and Biochemical Changes

#### 2.7.1. Chlorophyll and Chlorophyll a Fluorescence

**Figure 6:**
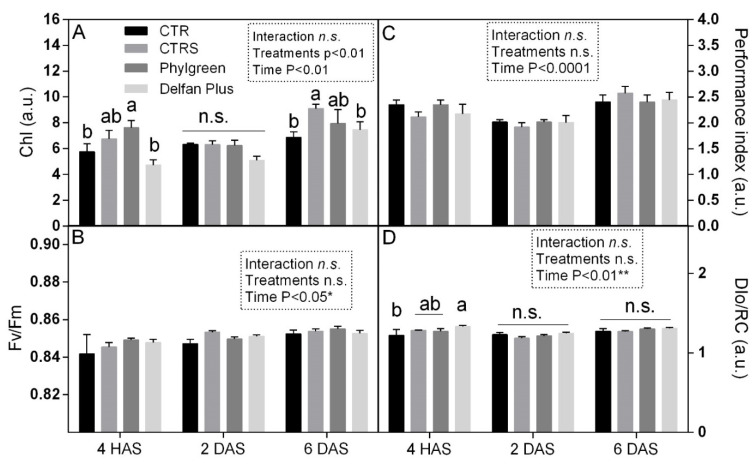
Non-destructive chlorophyll content (**A**), Fv/Fm ratio (**B**), performance index (**C**), and DIo/RC (**D**) measured in *Arabidopsis thaliana* plants treated in CTR, CTRS, Phylgreen, and Delfan Plus in 3 different moments (4 HAS, 2 and 6 DAS). Data are means with standard errors (*n* = 5). Statistical analysis was performed using two-way ANOVA and differences amongst means were determined using LSD test. Asterisks indicate significant differences, * for *p* < 0.05, ** for *p* < 0.01. In the A and D, significant differences for each timepoint have been highlighted using different letters.

The effect of treatments was evaluated by the determination of non-destructive chlorophyll content and chlorophyll a fluorescence measurements. Immediately 4 h after stress (HAS), the highest chlorophyll content was found in Phylgreen treatment, 7.6 arbitrary units (a.u.), while CTR showed a value of 5.8 a.u. The CTRS showed intermediate values, while CTR and Delfan Plus showed similar chlorophyll content. Two days after stress (DAS), the plants did not show significant variations among treatments. After 6 days of recovery, an increase of chlorophyll was observed in the CTRS and treated plants (Figure 6). Delfan Plus at the beginning of the experiments had lower chlorophyll but during recovery, it increased, reaching values similar to control and Phylgreen. The chlorophyll a fluorescence analysis, enables a non-destructive estimation of leaf functionality that can provide information on the health or stress conditions of plants. Statistical analysis showed that Fv/Fm, PI, and DIo/RC were not statistically different, while a difference was found in the time. The Fv/Fm ratio represents the maximum quantum efficiency of photosystem II and is an indicator of plant stress. The Fv/Fm ratio did not change among treatments, indicating that maximum quantum efficiency of PSII was not affected by the treatments (Figure 6B). The performance index (PI) represents the measurement of the leaf functionality of the plants. Phylgreen after 4 h heat stress exposure showed a value of 2.4 a.u. similar to CTR, indicating that the stress did not affect leaf functionality (Figure 6C). During the recovery period, the PI values ranged from 1.8 to 2.6 a.u. (Figure 6C). The dissipation energy per reaction centre (DIo/RC) indicates the energy lost as heat under stressful conditions. No differences among treatments were found (Figure 6D). 

#### 2.7.2. Total Phenolics and Anthocyanins

**Figure 7:**
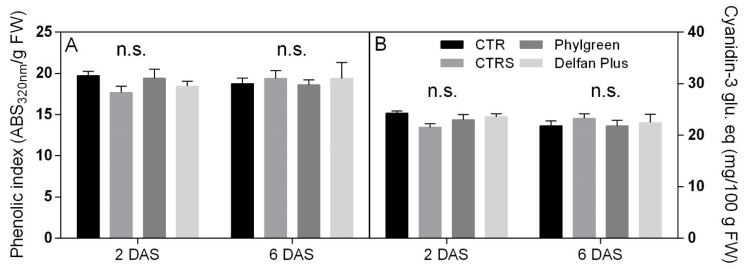
Total phenols expressed as phenolic index (**A**) and anthocyanin content (**B**) measured in *Arabidopsis thaliana* plants (*n* = 5) treated in CTR, CTRS, Phylgreen, and Delfan Plus. Data were subjected to two-way ANOVA and no differences were found.

Total phenols, expressed as the phenolic index, showed similar trends of anthocyanins for all the treatments. Data subjected to two-way ANOVA revealed that no significant differences were found. The phenolic index, after 2 days of recovery, ranged from 19.8 ABS_320nm_/g FW to 17.7 ABS_320nm_/g FW (Figure 7A).

Statistical analysis of anthocyanins showed that there were no significant differences among treatments. Anthocyanin concentrations ranged from 21.5 to 24 mg/100 g (Figure 7B).

## 3. Discussion

Biostimulants represent innovative tools to be used for protecting plants from abiotic stresses [15,22,23]. Several studies have shown that biostimulants can play a key role in helping crops against abiotic stresses, including heat stress [15]. A biostimulant containing a mixture of sugarcane molasses with yeast extract has been applied to tomatoes grown in high temperature (32 °C). The treated plants showed higher ascorbic acid in fruits and leaves. Positive effects were also found on yield [24]. The effect of treatments can be ascribed to the increase of the antioxidant compounds in plants that reduce the ROS accumulation. It is well known that ROS increase under heat stress and induce cell membrane damage with lipid peroxidation. Membrane destabilization, at leaf level, can reduce the electron transfer and the photosynthetic activity. At the physiological level, high temperatures can induce heat stress, while the severity of damage depends on crop sensitivity and exposure period. Heat stress is usually associated with a reduction in gas exchange and photosynthesis activity due to stomatal closure. The heat stress damage can affect the repair activity of photosystem II [25]. Chlorophyll *a* fluorescence measurement enables the non-destructive evaluation of plant health status and can provide useful information on the effect of treatments on heat stress tolerance induction. The Fv/Fm ratio and Fo have been successfully used as parameters for screening the tolerance of tropical fruit crops to heat stress [26]. Phylgreen treatment was the most active in increasing chlorophyll content under heat stress. This result can be explained by the effect of *Ascophyllum nodosum* extract that increases chlorophyll concentration and promotes growth [26]. After the recovery period, both biostimulants showed the same leaf functionality as non-stressed plants. Another mechanism used by plants to protect themselves from heat stress is the enhanced production of secondary metabolites and among them phenylpropanoids play a central role in plant response to heat stress [27,28,29]. Their antioxidant capacity is responsible for the neutralization of ROS generated by heat shock, thus preventing oxidative stress and cell damage [30]. The presence of secondary metabolites and ROS in controls and treated plants was also analyzed through histochemical analysis. Microscopic image analysis showed damage on the leaf blade in CTRS plants while Phylgreen and Delfan Plus treated plants avoided H_2_O_2_ accumulation, demonstrating the positive effect of biostimulants to mitigate heat stress. The Phylgreen is an Ascophyllum nodosum extract and the antioxidant activities demonstrated in plants and in vitro can explain the reduced electrolyte leakage [31]. In CTR leaves, microscopic analysis failed to detect any signal of ROS and secondary metabolites. The values of phenolic post-stress index demonstrated that plants were able to cope with heat stress and also enhanced the leafy functionality as observed by chlorophyll *a* fluorescence. 

Considering the analysis of cell dimension, the heat stress did not influence this parameter. In fact, the analysis of stomata dimensions showed no significant differences amongst the different samples and the slight differences observed in mesophyll cell dimension even if statistically significant, are more probably due to differences in dimension and developmental stage of the leaves analyzed than to the treatments. Considering all the histochemical analyses together, it could be observed that the Phylgreen and Delfan Plus treatments were shown to exert a positive effect on plant protection by reducing the consequences of thermal stress. The biostimulants induced a strong induction of differentially expressed transcription factors (TFs) compared to CTRS. Amongst the TFs associated with the heat responses, *AtZat10* and *AtZat12* were upregulated in both biostimulants with 2.4–2.7 and 2.7–3.0-fold change, respectively. The *AtZat* 10 is an HPS sensor of hydrogen peroxide and *AtZat12* is a heat response transcription factor [32], which suggests that the biostimulant treatment induced the activation of the transcriptional machinery involved in stress protection. Both biostimulants also induced the *AtHsfs*, which is associated with the heat response regulation of plants. Since these TFs were not expressed in the CTRS, this could be the strategy of the plants to enhance the activation of specific genes in heat defence. The common expression of *ATHsfs3* in both biostimulants has been found to induce thermotolerance in Arabidopsis [33]. *AtHSF3* can be considered as the key regulator of the immediate stress-induced activation of heat stress gene transcription [34]. These high gene regulation inductions have also been highlighted by the DAVID results which revealed the categories affected by the different treatments. DAVID bioinformatics tools can provide information on over-representation of the GO category terms [35] and help in understanding the mode of action of the biostimulants. In fact, most of the GO terms evidenced by the DAVID analysis were those related to the transcriptional regulation, abiotic stress defence and secondary metabolism. At the transcriptional level, among the heat stress-associated genes, the *AtHSP17.6A* was upregulated in all treatments. This gene was the highest expressed in CTRS and literature confirmed that it rapidly increases after heat stress, while it is not detectable in vegetative tissues in the absence of stress [36]. The activation of *AtHSP17.6A* is the plant strategy to counteract heat stress. Biostimulants were able to strongly induce a higher number of HSPs and it could indicate the mode of action of these products to enhance abiotic or heat stress tolerance. Phylgreen and Delfan Plus showed similar results in the activation of the heat shock proteins (HSP) DEGs. The most expressed DEG was *AtHSP17*.4 in both biostimulants and this gene encodes for the small HPS (sHPS) protein localized in the cytosol [37]. These HPS are mainly expressed during development, embryo maturation, and germination under normal conditions. In heat stress conditions, the expression of *AtHSP17.4* has been found in leaves and seems to have a protection function against the stress [36]. However, the expression of *AtHSP17.4* is associated with the activation of ABI3 [38,39]. In the current data presented, the expression of *AtABI3* was induced but at values from 0.5 to 0.8. Other highly expressed HPS genes were *AtHSP17.6 CII*, *AtHSP17.8 CI*, *AtHSP18.2*, and *AtHSP70* [40]. All these genes have been associated with different abiotic stresses. The *AtHSP17.6* has been induced by abscisic acid or osmotic stresses [37,41]. The expression of *AtHSP17*.8 is associated with drought and salt stress. Overexpression of *AtHSP17*.8 in Arabidopsis and lettuce increased tolerance against drought and high salinity stresses [42]. The expression of this gene seems to induce hypersensitivity to ABA with an improved crop sensitivity to water reduction. 

In this work the *AtHSP23*-5 was upregulated in both biostimulants suggesting an important role in heat stress defence. This gene is located in the nuclear genome and studied from evolution in Brassicaceae [43], while a mitochondrial *HPS22* has been found in *Drosophila melanogaster* under heat stress conditions [44].

## 4. Materials and Methods

### 4.1. Plant Materials and Treatments 

*Arabidopsis thaliana* L. (NASC ID: N6209, FRI-Sf2, http://arabidopsis.info/, (accessed on 6 March 2022)) plants were grown in fertilized peat substrate under controlled conditions (24 °C, 55–70 RH%, photoperiod 16/8 day/night 400 W m^−2^). In this study, we selected two commercially available products Phylgreen, and Delfan Plus. Phylgreen composition is the following: 1.2% p/p (1.3% p/v) mannitol, 2% p/p (2.2% p/v) alginic acid, 100% p/p (110% p/v) seaweed extract *Ascophyllum nodosum*, dry matter (from seaweed extract): 15% w/w (16.5% w/v). Delfan Plus is composed of L-α Free Amino Acids: 24.0% w/w 29.8% w/v, nitrogen (N), total: 9.0% w/w 11.2% w/v. Biostimulants were applied as priming treatments 12 h prior to heat stress as foliar application on plants at mature growing stage (stage 3.90, when the rosette growth is complete, https://www.arabidopsis.org/portals/education/growth.jsp, (accessed on 6 March 2022)) with concentration of 1 mL L^−1^ until run-off. From the growth chamber, control and treated plants were placed in a cabinet where intense heat stress (37 ± 1 °C) was applied for 4 h. The experimental scheme has been reported in Supplementary Figure S1. Each treatment sample was composed of 10 pots containing three plants per pot.

### 4.2. RNA Isolation

RNA sampling was immediately performed at the end of heat stress treatment, after 4 h of heat stress and 16 h after biostimulant application. Total RNA was extracted from 100 mg of leaf powder using the Spectrum Plant Total RNA Kit (Sigma-Aldrich, Milan, Italy). RNA purity and integrity were assessed with an Agilent 2100 bioanalyzer-RNA 6000 Nano Chip (Agilent Technologies, Santa Clara, CA, USA) and quantified with a Nanodrop 8000 (Thermo Scientific, Waltham, MA, USA). RNA samples with A260/A280 ≥ 1.9 and RNA integrity number (RIN) ≥ 7 were used for the RNA sequencing and further library preparation (libraries corresponding to a biostimulant product and to stressed and unstressed controls). 

### 4.3. RNA-Seq and Library Preparation 

Illumina sequencing and de novo assembly were performed at BMR Genomics Labs (Padua, Italy). The de novo assembly was performed to verify if the biostimulants could induce the activation of new specific genes. Random primed cDNA libraries were prepared with a TruSeq RNA Sample Prep kit (Illumina, San Diego, CA, USA) and sequenced in two paired-end modes in one run on the Illumina HiSeq-2000 Platform, generating up to 60M reads of 100 bp per sample. Alignment of reads to the reference genome of *Arabidopsis* (*Arabidopsis_thaliana* TAIR10 v42) was performed using HISAT 2.1.0 tool [45,46]), while gene association was performed with feature Counts v 1.6.0 (DESeq2 package version: 1.34.0 DESeq2 package version: 1.34.0). Specific libraries were prepared for unstressed untreated control plants (CTR) and for stressed untreated control plants (CTRS) and for biostimulants (applied on stressed plants). RNAseq data have been stored and available in the NCBI database (Accession n. PRJNA777374). The validation of RNAseq data has been performed by qPCR gene expression analysis on the following up- and downregulated genes *at2g29500*, *at5g12030*, *at5g12020*, *at1g09080*, *at3g46230*, *at1g72070*, *at5g59720*, *at5g37750*, *at5g37440*, and *at3g14200* (Supplementary Table S1) RPKM Log2 fold changes versus qPCR 2^−^^ΔΔ^**^CT^** log2 fold changes were compared as reported by Li [47] and correlation coefficient was *r* = 0.58. 

### 4.4. Bioinformatic Analysis

Gene annotation was performed using various platforms (including Blast2GO and Panther Tair tools). Genes differentially expressed were identified in each treatment and calculated as follows (Log2FC > 2, calculated as follow LOG ([A]; 2)-LOG ([B]; 2), A gene expression in the treatment vs. B gene expressed in the unstressed control) were selected and Venn diagrams were prepared to individuate common and unique responses in all the libraries. MapMan tool was used to display transcriptomic datasets onto diagrams of metabolic pathways and biological processes. This analysis allowed the identification of the most affected biochemical or signalling transduction pathways and the identification of the genes differentially expressed in the most interesting pathways for each treatment versus control unstressed/untreated. Enrichment of pathways, gene functions, and organelle associations based on Gene Ontology (GO) and other functional annotation data were identified using DAVID (http://david.abcc.ncifcrf.gov/, accessed 3 March 2021) [35]. The DAVID bioinformatics tool was also used to examine the biological significance of the transcriptome changes in the different treatments. Medium stringency was applied for the analyses. DAVID analysis identifies significantly enriched biological themes by examining for enrichment in over 40 different publicly available annotation categories (Supplementary Table S2), analyzing up- and downregulated sets separately. Significance was determined using a modified Fisher’s exact statistic (EASE score), and significantly enriched biological themes were identified as clusters of annotated terms and KEGG_PATHWAYs (https://www.genome.jp/kegg/pathway.html, accessed on 20 January 2022). A cluster enrichment score of 1.3 for an annotation cluster is equivalent to non-log scale 0.05, and therefore scores of 1.3 or greater are considered enriched [35] Fold-enrichment scores were also used to indicate the magnitude of enrichment for individual terms and KEGG_PATHWAYs, and fold-enrichment scores greater than 2 are suggestive of an informative change [35,46,47,48].

### 4.5. Non-Destructive Determination: Chlorophyll and Chlorophyll a Fluorescence 

Non-destructive analysis was carried out in vivo on leaf tissue for the leaf functionality evaluation of *Arabidopsis thaliana* plants after treatments and stress applications (12 h of priming and 4 h after stress—HAS, 2 days after stress—DAS, and 6 days after stress). The chlorophyll content was colorimetrically measured in vivo using a non-destructive instrument (CL-01, Hansatech, UK). Chlorophyll *a* fluorescence was measured using a portable fluorometer (Handy PEA, Hansatech, Kings Lynn, UK). Leaves were dark-adapted using leaf clips; after 30 min, a rapid pulse of high-intensity light of 3000 µmol m^−2^ s^−1^ (600 W m^−2^) was applied to the leaf inducing fluorescence. Fluorescence parameters were calculated automatically by the used device, such as Fv/Fm, the variable fluorescence to maximum fluorescence. Starting from these parameters, OJIP test analyses [49] were performed to determine the following index: performance index (PI); dissipation of energy per cross-section (DIo/RC) and density of reaction centre at P stage (RC/CSm).

### 4.6. Phenolic Index and Anthocyanins Content

These parameters were determined two and six days after stress application. The phenolic index and the total anthocyanin content in leaf tissue were determined using spectrophotometric methods (Spectrophotometer, Thermo, Italy). Phenolic index was determined by a direct measurement of the leaf extract absorbance at 320 nm. About 20–30 mg of fresh leaf tissue (disk of 5 mm diameter) was weighed and 3 mL methanolic HCl (1%) were added. After overnight incubation, the supernatant was read at 320 nm. The values were expressed as mg/100 g fresh weight (FW). Anthocyanin concentrations were determined on samples of 20–30 mg of fresh leaf (disks of 5 mm diameter) extract using 3 mL of methanolic HCl (1%). Samples were incubated overnight at 4 °C in darkness. The concentration of cyanidin-3-glucoside equivalents was determined spectrophotometrically at 535 nm using an extinction coefficient (ε) of 29,600. Sampling was performed taking four biological replicates for each treatment. 

### 4.7. Histochemical Analysis

Samples for the histochemical analysis were collected 4 h after heat stress. Arabidopsis leaves were detained in ethanol 100% and then observed using a Zeiss Axiophot D1 microscope, using ultraviolet epifluorescence (excitation filter 365 nm; dichroic mirror 395 nm, barrier filter 420 nm), equipped with an AxioCam MRc1 digital camera. Hydrogen peroxide (H_2_O_2_) detection was performed as previously reported [50]. Arabidopsis leaves were submerged in 3,3′-Diaminobenzidine (DAB) solution (1 mg/mL DAB, pH 3.8) and left overnight in the dark. The leaves were then rinsed in water, transferred to tubes containing ethanol 96% that were left in boiling water until chlorophyll was completely removed. The leaves were observed using a Zeiss Axiophot D1 microscope, equipped with an AxioCam MRc1 digital camera [51]. Cell death was quantified as electrolyte leakage measurement, as reported by Roberts et al. [52]. Briefly, for each leaf analyzed, 4 leaf discs (diameter: 8 mm) were floated in water for 30 min, then transferred to tubes containing 4 mL distilled water. The conductivity of the solution was determined with an Orion Conductivity Meter (µSiemens/cm). For each histochemical analysis, three biological replicates were used obtaining similar results. To determine mesophyll and stomata cell size, mature leaves were collected and treated with a clearing solution (160 g chloral hydrate, 20 mL glycerol in 60 mL water). Cleared leaves were mounted on slides, and interference contrast images were taken using a Zeiss IMAGE R.D1 microscope equipped with an AxioCam MRc1 digital camera. For each treatment, 3 plants were analyzed, from each plant one leaf was cleared and at least 50 mesophyll/stomata cells were measured. 

### 4.8. Statistical Analysis

Analytical data were subjected to two-way ANOVA performed by GraphPad Prism 6.0 (GraphPad Software, San Diego, CA, USA). Significant differences amongst means were determined using LSD multiple comparisons test. Specific details are also reported in figure legends. 

## 5. Conclusions

In conclusion, results revealed that biostimulants effectively induced activation of heat stress-associated genes belonging to different transcription factors and HSP families. The specific cluster of genes confirmed the physiological and biochemical data observed, demonstrating that the biostimulants were able to reduce the heat damage in Arabidopsis plants by activating antioxidant systems and heat repair systems. In general, both treatments with Phylgreen and Delfan Plus treatments had a positive effect in counteracting heat stress and transcriptional and biochemical data observed in *Arabidopsis thaliana* can represent a starting point for further studies focusing on agricultural crops.

## Figures and Tables

**Table 1:** Up- or downregulated (FC > 2) of differential expressed genes (DEGs) associated with heat stress in the CTRS, Phylgreen and Delfan Plus. Red bold color highlights DEGs common in all treatments, while blue bold color highlights DEGs common in Phylgreen, and Delfan Plus.

**Upregulated Genes**		
**Accession n**	**Description—CTRS**	**Upreg**
**at5g12030**	***Arabidopsis thaliana* Heat Shock Protein 17.6A**	**7**
**at1g71000**	**heat shock protein binding **	**2.807**
at4g24190	SHD (SHEPHERD), HSP90.7	2.585
**Description—Phylgreen**		
**at3g46230**	**AtHSP17.4**	**6.778**
**at5g12020**	**AtHSP17.6II (17.6 KDA CLASS II HEAT SHOCK PROTEIN)**	**5.775**
**at5g12030**	***Arabidopsis thaliana* Heat Shock Protein 17.6A**	**5.547**
**at2g29500**	**class I small heat shock protein (HSP17.6B-CI) 17.6 kDa**	**5.175**
**at1g07400**	**class I heat shock protein (HSP17.8-CI) 17.8 kDa**	**5.018**
**at2g21510**	**DNAJ heat shock N-terminal domain-containing protein **	4.64
**at1g72070**	**DNAJ heat shock N-terminal domain-containing protein **	**3.858**
**at5g51440**	**mitochondrial small heat shock protein (HSP23.5-M) 23.5 kDa **	**3.474**
**at5g59720**	**AtHSP18.2 (heat shock protein 18.2)**	**2.545**
at5g47600	heat shock protein-related	2.459
at1g44160	DNAJ chaperone C-terminal domain-containing protein	2.459
**at1g71000**	**heat shock protein binding **	**2.399**
**at3g12580**	***AtHSP70* (heat shock protein 70)**	**2.142**
**at5g52640**	**HSP81-1, ATHS83, HSP81.1, HSP83, ATHSP90.1**	**2.129**
**at1g28210**	**ATJ1; heat shock protein binding/nucleic acid binding **	**2.079**
at1g76770	heat shock protein-related	2
**Description—Delfan Plus**		
**at3g46230**	**AtHSP17.4**	**8.898**
**at5g12030**	***Arabidopsis thaliana* Heat Shock Protein 17.6A**	7.79
**at5g12020**	**AtHSP17.6II (17.6 KDA CLASS II HEAT SHOCK PROTEIN)**	7.562
**at2g29500**	**class I small heat shock protein (HSP17.6B-CI) 17.6 kDa**	6.98
**at5g59720**	**AtHSP18.2 (heat shock protein 18.2)**	6.523
**at1g07400**	**class I heat shock protein (HSP17.8-CI) 17.8 kDa**	5.938
**at1g72070**	**DNAJ heat shock N-terminal domain-containing protein**	4.585
**at5g51440**	**mitochondrial small heat shock protein (HSP23.5-M) 23.5 kDa **	4.281
at4g21320	HSA32 (HEAT-STRESS-ASSOCIATED 32);	4.138
**at5g52640**	**HSP81-1, ATHS83, HSP81.1, HSP83, ATHSP90.1|ATHSP90.1**	**3.262**
**at3g12580**	***AtHSP70* (heat shock protein 70)**	2.716
at2g32120	HSP70T-2 (HEAT-SHOCK PROTEIN 70T-2); ATP binding	2.413
at1g59860	17.6 kDa class I heat shock protein (HSP17.6A-CI)	2.409
at2g26150	*AtHSFA2*; DNA binding/transcription factor	2.369
**at2g21510**	**DNAJ heat shock N-terminal domain-containing protein**	2.259
**at1g71000**	**heat shock protein binding**	2.142
**at1g28210**	***AtJ1* ; heat shock protein binding **	**2.036**
**Downregulated genes**		
	**Description—Phylgreen**	**Downreg.**
at1g09080	AtBIP3; ATP binding	−2.518
at4g19590	DNAJ heat shock N-terminal domain-containing protein	−2
at2g03020	heat shock protein-related	−2

**Table 2:** DAVID functional analysis: functional annotation chart (FACH) of differentially expressed genes in CTRS, Phylgreen, and Delfan Plus (FC > 2) recognized in DAVID database. Functional category, terms, *p* value, fold enrichment, and statistical significance (Bonferroni, Benjamini, and FDR). Top ten (if available) genes in each treatment.

**CTRS**					
**Category**	**Terms**	***p***	**F**	**Bonferroni**	**FDR**
INTERPRO	IPR003614: Knottin, scorpion toxin-like	3.1 × 10^11^	7.4 × 10^14^	2.2 × 10^15^	4.8 × 10^16^
INTERPRO	IPR002472: Palmitoyl protein thioesterase	6.3 × 10^11^	2.1 × 10^15^	4.0 × 10^15^	9.6 × 10^15^
GOTERM_MF_DIRECT	GO:0016790~thiolester hydrolase activity	6.5 × 10^11^	2.1 × 10^15^	2.3 × 10^16^	9 × 10^15^
GOTERM_MF_DIRECT	GO:0008474~palmitoyl-(protein) hydrolase activity	7.7 × 10^11^	1.1 × 10^16^	2.6 × 10^15^	1.1 × 10^16^
SMART	SM00505:Knot1	2.7 × 10^12^	9.9 × 10^15^	3.1 × 10^15^	3.0 × 10^16^
GOTERM_BP_DIRECT	GO:0002084~protein depalmitoylation	6.1 × 10^14^	1.0 × 10^16^	9.8 × 10^15^	8.7 × 10^15^
UP_SEQ_FEATURE	active site:Proton acceptor	6.6 × 10^15^	1.7 × 10^16^	9.8 × 10^15^	9.3 × 10^15^
GOTERM_CC_DIRECT	GO:0005829~cytosol	7.0 × 10^15^	1.3 × 10^16^	7.4 × 10^15^	8.4 × 10^15^
INTERPRO	IPR008176:Gamma thionin	8.4 × 10^15^	9.3 × 10^15^	10 × 10^15^	12 × 10^16^
KEGG_PATHWAY	ath00062:Fatty acid elongation	3.9 × 10^16^	5.6 × 10^15^	3.0 × 10^15^	4.2 × 10^15^
**Phylgreen**					
**Category**	**Term**	***p***	**FE**	**Bonf.**	**FDR**
GOTERM_BP_DIRECT	GO:0009611~response to wounding	4.1 × 10^−19^	5.2	2.7 × 10^−16^	6.1 × 10^−16^
GOTERM_BP_DIRECT	GO:0006952~defense response	6.53 × 10^−19^	3.0	4.2 × 10^−16^	9.7 × 10^−16^
UP_SEQ_FEATURE	DNA-binding region: AP2/ERF	1.2 × 10^−12^	5.1	8.1 × 10^−10^	1.8 × 10^−9^
INTERPRO	IPR001471:AP2/ERF domain	1.5 × 10^−11^	4.7	1.2 × 10^−8^	2.3 × 10^−8^
GOTERM_BP_DIRECT	GO:0010200~response to chitin	2.2 × 10^−13^	5.3	1.4 × 10^−10^	3.3 × 10^−10^
GOTERM_BP_DIRECT	GO:0009753~response to jasmonic acid	4.7 × 10^−13^	4.8	3.1 × 10^−10^	7 × 10^−10^
SMART	SM00380:AP2	3. × 10^−11^	4.4	4.5 × 10^−9^	4.1 × 10^−8^
INTERPRO	IPR016177:DNA-binding, integrase-type	1.1 × 10^−10^	4.3	8.5 × 10^−8^	1.6 × 10^−7^
GOTERM_MF_DIRECT	GO:0043565~sequence-specific DNA binding	3.9 × 10^−9^	2.3	1.7 × 10^−6^	5.5 × 10^−6^
GOTERM_BP_DIRECT	GO:0006355~regulation of transcription, DNA-templated	143	11.5	1.3 × 10^−8^	1.6 × 10^−5^
**Delfan Plus**					
**Category**	**Term**	***p***	**FE**	**Bonf.**	**FDR**
GOTERM_MF_DIRECT	GO:0003700 transcription factor activity, sequence-specific DNA binding	1.7 × 10^08^	2.66 × 10^−16^	2.66 × 10^−16^	6.12 × 10^−16^
GOTERM_BP_DIRECT	GO:0042542 response to hydrogen peroxide	1.8 × 10^−9^	4.24 × 10^−16^	2.1 × 10^−16^	9.74 × 10^−16^
UP_SEQ_FEATURE	region of interest: type E motif	1.2 × 10^−8^	1.45 × 10^−10^	4.83 × 10^−11^	3.33 × 10^−10^
GOTERM_BP_DIRECT	GO:0009408 response to heat	2. × 10^−10^	3.06 × 10^−10^	7.64 × 10^−11^	7.02 × 10^−10^
UP_KEYWORDS	Pyrrolidone carboxylic acid	2.3 × 10^−12^	8.16 × 10^−10^	8.16 × 10^−10^	1.80 × 10^−9^
UP_SEQ_FEATURE	DNA-binding region:WRKY	5.9 × 10^−9^	3.55 × 10^−9^	3.55 × 10^−9^	1.17 × 10^−8^
GOTERM_MF_DIRECT	GO:0044212 transcription regulatory region DNA binding	6.7 × 10^−9^	1.21 × 10^−8^	1.21 × 10^−8^	2.31 × 10^−8^
UP_SEQ_FEATURE	region of interest:Type E(+) motif	8.1 × 10^−9^	4.52 × 10^−9^	4.52 × 10^−9^	4.13 × 10^−8^
UP_KEYWORDS	Apoplast	1.1 × 10^−11^	8.50 × 10^−8^	4.25 × 10^−8^	1.63 × 10^−7^
GOTERM_BP_DIRECT	GO:0009751 response to salicylic acid	2.0 × 10^−10^	1.67 × 10^−6^	8.33 × 10^−7^	5.50 × 10^−6^

**Table 3:** DAVID functional analysis: functional annotation cluster (FAC) of differentially expressed genes in CTRS, Phylgreen, and Delfan Plus (Enrichment Score >2) recognized in DAVID database. Functional category, terms, *p* value, fold enrichment, and statistical significance (Bonferroni, Benjamini, and FDR). In the table, two annotation clusters (if available) with highest enrichment score for each treatment.

**CTRS**							
**Annotation Cluster 1—Enrichment Score: 2.2**							
**Category**	**Term**	**%**	***p***	**FE**	**Bonf**	**Benj**	**FDR**
INTERPRO	IPR002472: Palmitoyl protein thioesterase	0.5	6.3 × 10^−4^	21.2	0.40	0.22	0.96
GOTERM_MF_DIRECT	GO:0016790~thiolester hydrolase activity	0.5	6.5 × 10^−4^	20.9	0.20	0.22	0.90
GOTERM_MF_DIRECT	GO:0008474~palmitoyl-(protein) hydrolase activity	0.7	7.7 × 10^−4^	11.4	0.26	0.14	1.06
KEGG_PATHWAY	ath00062: Fatty acid elongation	0.8	0.004	5.6	0.30	0.30	4.15
GOTERM_BP_DIRECT	GO:0002084~protein depalmitoylation	0.5	0.006	10.3	0.98	0.98	8.71
KEGG_PATHWAY	ath01212: Fatty acid metabolism	0.7	0.17	2.2	1.0	0.93	88.32
**Phylgreen**							
**Annotation Cluster 1—Enrichment Score: 8.6**							
**Category**	**Term**	**%**	***p***	**FE**	**Bonf.**	**Benj.**	**FDR**
UP_SEQ_FEATURE	DNA-binding region:AP2/ERF	2.4	1.1 × 10^−12^	5.1	8.1 × 10^−10^	8.1 × 10^−10^	1.8 × 10^−9^
INTERPRO	IPR001471:AP2/ERF domain	2.4	1.5 × 10^−11^	4.7	1.2 × 10^−8^	1.2 × 10^−8^	2.3 × 10^−8^
SMART	SM00380:AP2	2.4	3.6 × 10^−11^	4.4	4.5 × 10^−9^	4.5 × 10^−9^	4.1 × 10^−8^
INTERPRO	IPR016177:DNA-binding, integrase-type	2.4	1.1 × 10^−10^	4.3	8.5 × 10^−8^	4.2 × 10^−8^	1.6 × 10^−7^
UP_KEYWORDS	Ethylene signalling pathway	1.9	8.3 × 10^−7^	3.3	2.0 × 10^−4^	5.0 × 10^−5^	0.001
GOTERM_BP_DIRECT	GO:0009873~ethylene-activated signalling pathway	1.9	2.1 × 10^−6^	3.2	0.001	1.3 × 10^−4^	0.003
UP_KEYWORDS	Activator	3.5	6.9 × 10^−6^	2.1	0.001	3.3 × 10^−4^	0.008
**Annotation Cluster 2—Enrichment Score: 6.3**							
**Category**	**Term**	**%**	***p***	**FE**	**Bonf.**	**Benjamini**	**FDR**
GOTERM_MF_DIRECT	GO:0003700~transcription factor activity, sequence-specific DNA binding	10.3	8.3 × 10^−12^	1.8	3.6 × 10^−9^	3.6 × 10^−9^	1.2 × 10^−8^
GOTERM_MF_DIRECT	GO:0043565~sequence-specific DNA binding	5.0	3.9 × 10^−9^	2.3	1.7 × 10^−6^	8.3 × 10^−7^	5.5 × 10^−6^
GOTERM_BP_DIRECT	GO:0006355~regulation of transcription, DNA-templated	11.6	1.3 × 10^−8^	1.6	8.5 × 10^−8^	1.7 × 10^−6^	2.0 × 10^−5^
GOTERM_BP_DIRECT	GO:0006351~transcription, DNA-templated	10.5	2.1 × 10^−8^	1.6	1.4 × 10^−5^	2.3 × 10^−6^	3.2 × 10^−5^
UP_KEYWORDS	Transcription regulation	10.7	1.2 × 10^−7^	1.6	2.9 × 10^−5^	1.5 × 10^−5^	1.6 × 10^−4^
UP_KEYWORDS	Transcription	10.7	4.5 × 10^−7^	1.6	1.1 × 10^−4^	3.6 × 10^−5^	5.8 × 10^−4^
UP_KEYWORDS	DNA-binding	8.9	8.4 × 10^−5^	1.4	0.02	0.003	0.11
GOTERM_MF_DIRECT	GO:0003677~DNA binding	8.9	0.002	1.3	0.49	0.109	2.26
**Delfan Plus**							
**Annotation Cluster 1—Enrichment Score: 5.5**							
**Category**	**Term**	**%**	***p***	**FE**	**Bonf.**	**Benj.**	**FDR**
GOTERM_BP_DIRECT	GO:0042542~response to hydrogen peroxide	1.2	1.9 × 10^−7^	7.1	1.1 × 10^−4^	5.7 × 10^−5^	2.8 × 10^−4^
GOTERM_BP_DIRECT	GO:0009408~response to heat	2.0	2.0 × 10^−6^	3.7	0.001	4.2 × 10^−4^	0.003
GOTERM_BP_DIRECT	GO:0009644~response to high light intensity	1.0	8.6 × 10^−5^	5. 4	0.05	0.007	0.12
**Annotation Cluster 2—Enrichment Score: 4.5**							
**Category**	**Term**	**%**	***p***	**FE**	**Bonf.**	**Benj.**	**FDR**
UP_SEQ_FEATURE	DNA-binding region:WRKY	1.1	5.9 × 10^−6^	5.7	0.003	0.001	0.009
SMART	SM00774:WRKY	1.2	2.6 × 10^−5^	4.9	0.003	0.003	0.03
INTERPRO	IPR003657:DNA-binding WRKY	1.2	3.5 × 10^−5^	4.0	0.02	0.02	0.05

## Data Availability

Data of RNAseq are available on NCBI, accession number PRJNA777374.

## Supplementary Materials

The following supporting information can be downloaded at: https://www.mdpi.com/article/10.3390/plants11091130/s1, Figure S1: Experimental plan; Figure S2: Category and terms reported in the DAVID analysis. Table S1: Set of primers used for the qPCR; Table S2: Terms and categories used in the DAVID analysis. Supplementary Dataset S1; Supplementary Dataset S2; Supplementary Dataset S3.
