# Supplementary material for: Priming Treatments with Biostimulants to Cope the Short-Term Heat Stress Response: A Transcriptomic Profile Evaluation
Source: Plants (Basel). 2022 Apr 21;11(9):1130. doi: 10.3390/plants11091130 (PMC9101846; doi:10.3390/plants11091130)
Supplement: Supplementary file 1 [file plants-11-01130-s001.zip › Supplementary Figure S2.pdf]

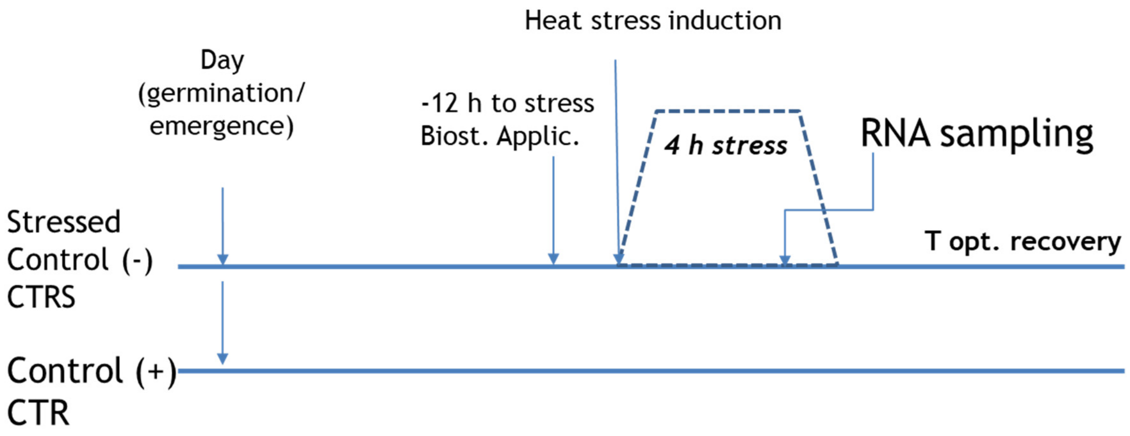

Supplementary Figure S2: Plants were grown without stress until the rosette stage. Products were applied on plants (Phylgreen and Delfan Plus) and 12 hours later heat stress started to increase rapidly until 37° C. Plants were exposed at 37° for 4 hours, then leaves were sampled for RNA extraction.
