# Supplementary material for: Priming Treatments with Biostimulants to Cope the Short-Term Heat Stress Response: A Transcriptomic Profile Evaluation
Source: Plants (Basel). 2022 Apr 21;11(9):1130. doi: 10.3390/plants11091130 (PMC9101846; doi:10.3390/plants11091130)
Supplement: Supplementary file 1 [file plants-11-01130-s001.zip › Supplementary Table S1.pdf]

Suppl. Table S1. Set of primers used for RNAseq validation using qPCR.

**at2g29500 17.6 kDa class I small heat shock protein (HSP17.6B-CI)**						
	**Template strand**	**Length**	**Start**	**Stop**	**Tm**	**GC%**
at2g29500 For	ACGAGGAGGTTTAGGTTGCC	Plus	20	325	344	59.68
at2g29500 Rev	ACACACCATTCTCCATCGCA	Minus	20	394	375	59.68
**at5g12030 AT-HSP17.6A, HSP17.6 | AT-HSP17.6A (ARABIDOPSIS THALIANA HEAT SHOCK PROTEIN 17.6A)**						
**at5g12030 For**	GACATGCTTGAAGCCCCTGA	Plus	20	46	65	60.32
**at5g12030 Rev**	ATGTCCACGGCGAAAACGTA	Minus	20	188	169	60.32
**at5g12020 Symbols: HSP17.6II | HSP17.6II (17.6 KDA CLASS II HEAT SHOCK PROTEIN)**						
**at5g12020 For**	AAGTCCCCGAAGACCACAAC	Plus	20	285	304	59.89
**at5g12020 Rev**	ATTGCCTTAGCGTCTCGCAT	Minus	20	360	341	60.18
**at1g09080 Symbols: BIP3 | BIP3; ATP binding | chr1:2929217-2931841**						
**at1g09080 For**	TTTCTGACTGCTTTGATGTCAAGT	Plus	24	110	133	59.12
**at1g09080 Rev**	GATCCCTATCACCGTCCCCA	Minus	20	181	162	60.47
**at3g46230 ATHSP17.4, HSP17.4 | ATHSP17.4 | chr3:16984118-16984841**						
**at3g46230 For**	GGCGAGTATGGAGAATGGGG	Plus	20	489	508	59.96
**at3g46230 Rev**	GGACTTGACCTCCGGCTTAC	Minus	20	561	542	60.11
**at1g72070 DNAJ heat shock N-terminal domain-containing protein | chr1:27118621-27119708**						
**at1g72070 For**	CCGCTTACGAGTGTTTGATGC	Plus	21	558	578	60.20
**at1g72070 Rev**	TCCCATCCATTCCTCCCACT	Minus	20	655	636	59.95
**at5g59720 Symbols: HSP18.2 | HSP18.2 (heat shock protein 18.2) | chr5:24062577-24063277**						
**at5g59720 For**	ATAAGTGGCACCGTGTGGAG	Plus	20	614	633	60.04
**at5g59720 Rev**	TTGAGGCTTCTTCTCAGGCG	Minus	20	762	743	60.04
**at5g37750 heat shock protein binding / unfolded protein binding | chr5:14995789-14997046**						
**at5g37750 For**	ACTGTGAACTAAGAAGTACGAGTCA	Plus	25	1026	1050	59.47
**at5g37750 ev**	AAAGGAGAACCCGCTCTTGT	Minus	20	1174	1155	59.23

**at5g37440 DNAJ heat shock N-terminal domain-containing protein | chr5:14844359-14845560**						
**at5g37440 For**	GGCGAGGACATATGTGAGCTA	Plus	21	1154	1174	59.39
**at5g37440 Rev**	ACAAGACACACTCCTACACATTGA	Minus	24	1284	1261	59.90
**at3g14200 DNAJ heat shock N-terminal domain-containing protein | chr3:4712574-4714653**						
**at3g14200 For**	ACAATGTTCCATCGGCTGGT	Plus	20	966	985	59.96
**at3g14200 Rev**	GAAGTGGCTATGAGGTGGGG	Minus	20	1037	1018	59.82

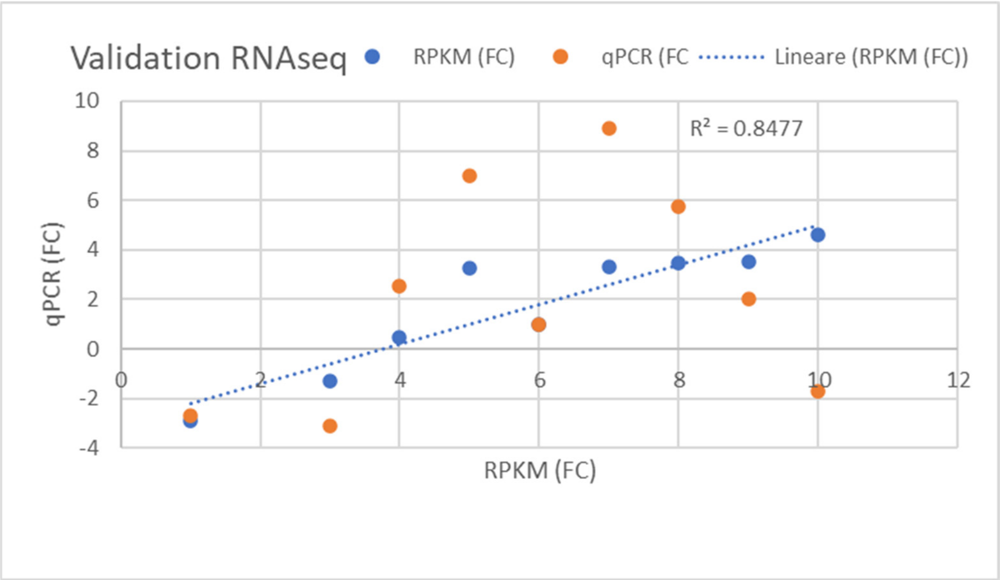

Linear regression between the RNAseq data versus qPCR data both are expressed as Log2 fold change of $2^{-\Delta\Delta CT}$. The correlation coefficient was r=0.58.
