# Supplementary material for: Priming Treatments with Biostimulants to Cope the Short-Term Heat Stress Response: A Transcriptomic Profile Evaluation
Source: Plants (Basel). 2022 Apr 21;11(9):1130. doi: 10.3390/plants11091130 (PMC9101846; doi:10.3390/plants11091130)
Supplement: Supplementary file 1 [file plants-11-01130-s001.zip › Supplementary Table S2.pdf]

Suppl. Table S2. Terms and categories used in the DAVID analysis.

Term	Meaning and links to databases
Functional Annotation Chart (FACH)	Identify enriched annotation terms associated with user's gene list
Functional Annotation Clustering (FAC)	Cluster functionally similar terms associated with user's gene list into groups
Categories: database used for annotation and clustering with similar biological meaning	INTERPRO: https://www.ebi.ac.uk/interpro/<br>GOTERM: http://geneontology.org/<br>SMART: http://smart.embl-heidelberg.de/<br>UNIPROT UP_SEQ_FEATURE: https://www.uniprot.org/help/sequence_annotation<br>UNIPROT UP_KEYWORDS: https://www.uniprot.org/help/keywords
