# Supplementary figures and images for: Priming Treatments with Biostimulants to Cope the Short-Term Heat Stress Response: A Transcriptomic Profile Evaluation
Source: Plants (Basel). 2022 Apr 21;11(9):1130. doi: 10.3390/plants11091130 (PMC9101846; doi:10.3390/plants11091130)

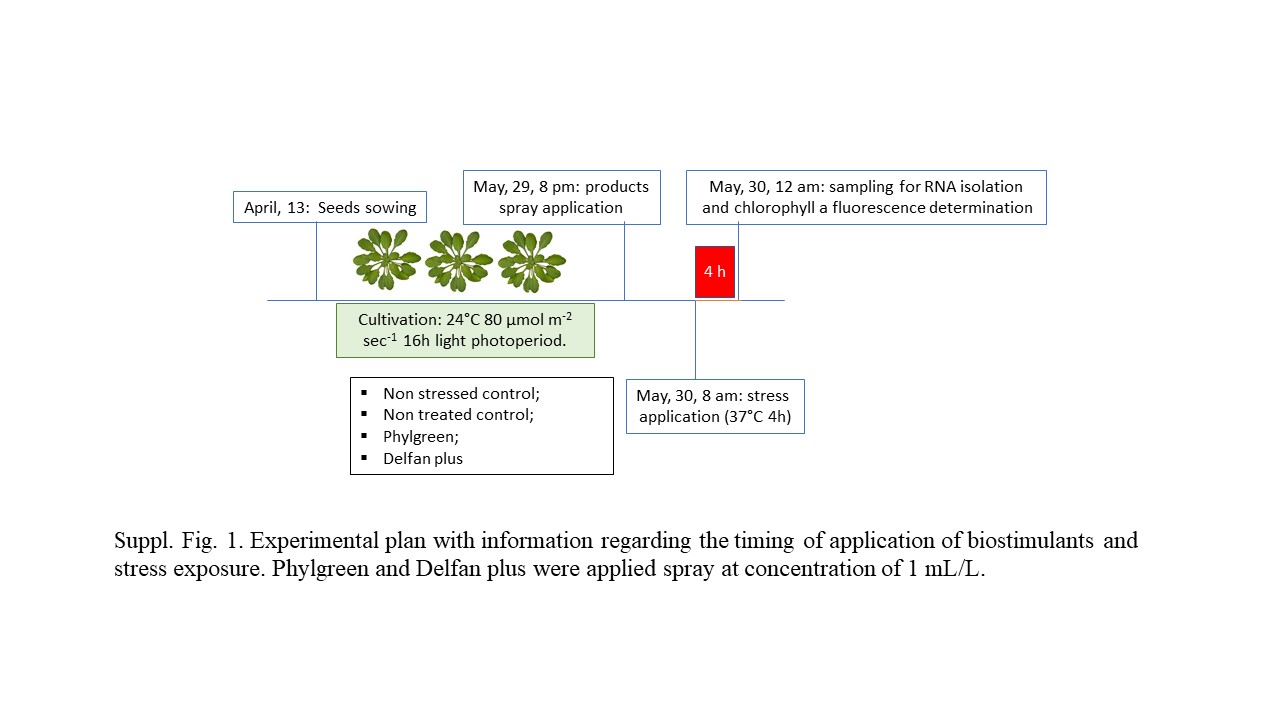

Supplement: Supplementary file 1 [file plants-11-01130-s001.zip › Supplementary Figure S1.jpg]
